# Validation of an LC–MS/MS Method for Urinary Cortisol in Dogs and Reference Interval, with an Exploratory Comparison to Immunoassay

**DOI:** 10.3390/ani15182682

**Published:** 2025-09-13

**Authors:** Tommaso Furlanello, Francesca Maria Bertolini, Luca Magna, Jose Sanchez del Pulgar, Riccardo Masti

**Affiliations:** 1 San Marco Veterinary Clinic and Laboratory, Via dell’Industria 3, 35030 Veggiano, Italy; tf@sanmarcovet.it (T.F.); francesca.bertolini@sanmarcovet.it (F.M.B.); luca.magna@sanmarcovet.it (L.M.); 2 CREA Research Centre for Food and Nutrition, Via Ardeatina 546, 00178 Rome, Italy; jose.sanchezdelpulgar@crea.gov.it

**Keywords:** canine endocrine disorders, veterinary endocrinology, targeted mass spectrometry, hormone measurement

## Abstract

Cortisol measurement in dogs commonly supports the diagnosis of hypercortisolism or hypoadrenocorticism. Unlike serum cortisol, urine cortisol is less affected by daily fluctuations, and collection is non-invasive, making the urine cortisol-to-creatinine ratio (UCCR) a useful diagnostic tool. Immunoassays are commonly used but lack specificity for free, biologically active cortisol. Liquid chromatography–tandem mass spectrometry (LC-MS/MS) is recommended to overcome this limitation. This study aimed to validate an LC-MS/MS method for urinary cortisol measurement, establish a reference interval for UCCR in dogs, and compare the results with the commercial immunoassay system Immulite^®^ 2000 Xpi (Siemens, Cary, NC, USA). A comprehensive method validation was performed. Across different concentrations, the intra- and inter-assay coefficients of variation (CV) ranged from 2.39% to 9.45%, accuracy ranged from 87.7% to 105.5%, and bias varied from −12.3% to 5.53%, which were all within acceptable limits. The method demonstrated excellent linearity (r^2^ ≥ 0.98), and stability under various conditions was satisfactory (RSD: −12.4% to 13.6%). Interference from endogenous and exogenous corticosteroids was negligible. The established reference interval for UCCR was 0.21–2.84 × 10^−6^. The exploratory comparison with the immunoassay revealed systematic and proportional differences between methods. The LC-MS/MS method demonstrated excellent analytical performance in terms of accuracy and specificity, and showed suitability for clinical application.

## Simple Summary

The urinary cortisol measurement is a valuable tool in diagnosing adrenal disorders in dogs, offering advantages over serum testing due to its stability and non-invasive collection. Traditional immunoassays lack specificity for free cortisol, the active form. This study focused on validating a precise LC-MS/MS method compared with the commercial immunoassay system Immulite^®^ 2000 Xpi. The validation confirmed the method’s reliability, showing strong precision and accuracy, with negligible interference from corticoids. A reference range for the urine cortisol-to-creatinine ratio (UCCR) was also established. These findings highlight the LC-MS/MS technique as a robust and clinically useful approach for assessing urinary cortisol in dogs.

## 1. Introduction

Cortisol measurement is a common practice in canine medicine, mainly used to diagnose hypercortisolism and to monitor both medical and surgical treatments. It also plays a key role in diagnosing hypoadrenocorticism and in assessing stress-related conditions. However, a single resting or basal plasma or serum cortisol determination has limited diagnostic value due to overlapping cortisol concentrations between healthy and diseased animals, including those with adrenal and non-adrenal illnesses [1].

To address this, dynamic tests, such as the ACTH stimulation test (ACTHst) and the low-dose dexamethasone suppression test (LDDST), are commonly employed in clinical practice. The LDDST, although time-consuming, has high diagnostic sensitivity (85–97%) but lower diagnostic specificity (67–81%) when evaluating dogs with consistent clinical signs. The ACTHst is a safe and time-efficient screening test for hypercortisolism, with moderate diagnostic sensitivity, although this is significantly lower (33–63%) in dogs affected by adrenal-dependent hypercortisolism [2,3].

Cortisol and its metabolites are excreted in urine. Measuring urinary corticoids in a morning sample captures cortisol release over several hours, thereby accounting for fluctuations in plasma corticoid concentrations. Expressing urinary corticoids concentration relative to creatinine concentration (urine cortisol-to-creatinine ratio, UCCR) accounts for differences in urine concentration and provides an indirect assessment of circulating cortisol concentrations over time [1]. The UCCR has been widely used to screen dogs for hyperadrenocorticism due to its convenience and availability through commercial diagnostic laboratories. Initially considered highly sensitive for detecting excess circulating cortisol, recent studies have challenged this assumption, reporting only modest specificity and sensitivity (80.4%) and a low negative predictive value (56.8%) [4]. A major limitation of the UCCR test is its inability to distinguish between free cortisol and its metabolites, which are abundant in urine. Clinical laboratories uniformly employ antibodies that cannot differentiate between free cortisol and most of its metabolites, making this analytical specificity issue more problematic in urine samples [5]. The Immulite^®^ 2000 Siemens immunoassay is the most commonly used method, which has been validated in veterinary medicine; however, the type of anti-cortisol antibody used is not disclosed and may have evolved over time, necessitating adjustments to reference ranges and clinical cut-offs [4,5,6].

These limitations are well documented in human medicine. While widely used due to their relatively low cost and ease of use, the efficiency of immunoassays can be compromised by cross-reactivity with other steroid hormones and is highly dependent on antibody quality [7]. Urine contains unconjugated urinary free cortisol (UFC), as well as conjugated cortisol, along with other abundant cortisol metabolites, both conjugated and unconjugated, with chemical structures similar to cortisol. These metabolites may cross-react with immunoassay antibodies, interfering with UFC measurements [8].

Given these limitations of traditional immunoassay-based approaches, alternative techniques such as mass spectrometry have gained increasing attention, offering high analytical sensitivity and specificity, as well as rapid sample preparation. In humans, LC-MS/MS methods have been proposed for diagnosing various pathologies involving endogenous corticosteroids. In fact, LC-MS/MS is considered the analytical method of choice for endogenous and exogenous corticosteroid measurements, representing the current gold standard for clinical cortisol measurement [9]. LC-MS/MS is known to provide a powerful technique for molecular characterization, representing the best available tool for characterizing free cortisol levels. LC-MS/MS is recognized as the gold standard for analyzing cortisol in different bodily fluids, capable of distinguishing between various steroid species and even synthetic compounds [7,8,9,10].

Although mass spectrometry has been rarely applied in canine medicine, Galeandro et al. used gas chromatography–mass spectrometry (GC-MS) as a reference method to evaluate five different immunoassays [11]. Others have employed the same technique to evaluate cortisol metabolites [12,13], while the more advanced LC-MS/MS has been used to assess cortisoluria in dogs treated with trilostane [14], an area where UCCR failed to reflect treatment efficacy. The inability to evaluate trilostane monitoring using UCCR is typically attributed to the analytical method, which detects both cortisol and its metabolites, many of which are altered by this steroidogenesis inhibitor [15].

The objective of this study was to validate an LC–MS/MS method for measuring urinary cortisol in dogs according to EMA guidelines, establish a reference interval for UCCR, and compare results with those obtained using a commercial immunoassay system (Immulite^®^ 2000 Xpi).

## 2. Materials and Methods

### 2.1. Urine Sample Collection

Residual urine samples were obtained from client-owned dogs presented to the San Marco Veterinary Clinic and Diagnostic Laboratory (Veggiano, Italy) for routine wellness visits. Forty-eight samples were purposively selected to establish reference intervals for urinary cortisol concentration in clinically healthy adult dogs. The animals that were included in the study were undergoing routine procedures, such as preoperative evaluations for neutering or dental procedures, oestrous cycle monitoring, or general health screening. Health status was confirmed based on a complete clinical evaluation, including a review of the animal’s medical history, physical examination, complete blood count, serum biochemistry profile, and urinalysis. Only dogs with no clinical signs or laboratory evidence of endocrine or systemic disorders known to influence glucocorticoid metabolism were included. Urine samples were collected by either spontaneous voiding or cystocentesis, immediately aliquoted into polypropylene tubes, and stored at −20 °C for no longer than two months. All samples were thawed only once, immediately prior to analysis.

### 2.2. Reagents and Materials

LC-MS-grade methanol was procured from Merck (LiChrosolv^®^, Darmstadt, Germany). Certified reference standards for cortisol (>99.9% purity), D4-cortisol, along with sodium phosphate dibasic, were sourced from Sigma-Aldrich (Merck, Darmstadt, Germany). Ammonium fluoride was obtained from Supelco (Merck, Bellefonte, PA, USA). Cortisone, trilostane, methylprednisolone, and dexamethasone standards (Merck, Darmstadt, Germany) were used in selectivity tests.

### 2.3. LC-MS/MS Instrumentation and Chromatographic Conditions

Quantitative analysis was conducted using an Agilent 1260 Infinity liquid chromatograph coupled to a 6490 Triple Quadrupole mass spectrometer (Agilent Technologies, Santa Clara, CA, USA) equipped with an electrospray ionization (ESI) source operating in positive ion mode. Chromatographic separation was achieved on a Phenomenex Luna^®^ Omega Polar C18 column (100 × 2.1 mm, 3 µm particle size; Phenomenex, Vaerloese, Denmark) maintained at 50 °C. The mobile phase consisted of 0.05 mM ammonium fluoride in water (A) and methanol (B), delivered at a flow rate of 0.4 mL/min. A gradient elution program was applied as follows: 45% B from 0 to 0.5 min, linearly increasing to 57% B by 4.5 min, then ramping to 100% B from 4.51 to 5.51 min, followed by re-equilibration to initial conditions. The total run time was 8 min, with an injection volume of 2 µL. Compounds were detected using the Multiple Reaction Monitoring (MRM) mode. The instrument parameters were set as follows: drying gas temperature, 180 °C; drying gas flow rate, 16 L/min; nebulizer pressure, 30 psi; sheath gas temperature, 250 °C; sheath gas flow rate, 12 L/min. Cortisol was monitored using the transitions *m*/*z* 363.2→121.0, 97.0, and 309.2 with collision energies (CE) ranging from 18 to 28 eV and a cell acceleration voltage (CAV) of 5 V. The internal standard, cortisol-D4, was monitored at *m*/*z* 367.2→121.0, 273.1 (CE: 18 eV; CAV: 5 V). Data acquisition and quantitative analysis were performed using Agilent MassHunter software (version 6.0). Selectivity testing was carried out by monitoring the following analytes and MRM transitions: cortisone (*m*/*z* 361.2→163.1, 121.2; CE: 25 eV; CAV: 5 V), dexamethasone (*m*/*z* 393.5→149, 167; CE: 20 eV; CAV: 5 V), methylprednisolone (*m*/*z* 375.1→161.1, 135.0; CE: 15 eV; CAV: 5 V), and trilostane (*m*/*z* 330.5→263.0, 245.0; CE: 15 eV; CAV: 5 V).

### 2.4. Quantification of Urinary Creatinine and Immunological Urinary Cortisol

Urinary creatinine (uCr) concentrations were measured using the Atellica^®^ CH Analyser (Siemens Healthcare Diagnostics Inc., Duisburg, Germany), employing the Creatinine_2 (Crea_2) assay [16], which is based on a kinetic modification of the Jaffé method. In this assay, creatinine reacts with alkaline picrate to form a colored complex, and the rate of absorbance change is proportional to the creatinine concentration. The method includes correction algorithms to reduce interference from non-creatinine chromogens. The analytical measurement range for urine samples is 3.00 to 245.00 mg/dL, with a limit of quantification (LOQ) of 3.00 mg/dL. For the method comparison study, the same samples were also analyzed on an Immulite^®^ 2000 Xpi (Siemens, Cary, NC, USA) using a cortisol immunoassay (Cortisol, Immulite 2000 XPi, Siemens, Tarrytown, NY, USA) with kit lot numbers greater than 550. This kit was originally developed for serum cortisol measurement in humans and was previously adapted for use in canine urine [5]. All analyses were performed according to the manufacturer’s protocols, and appropriate internal quality control procedures were followed.

### 2.5. Sample Processing

Aliquots of urine (100 µL) were transferred into 1.5 mL polypropylene microcentrifuge tubes and combined with 100 µL of 50 mM sodium phosphate dibasic buffer (pH 7.4) containing deuterated internal standards (D4-cortisol, 3 ng/mL final concentration). The samples were vortex-mixed for 5 min to ensure complete homogenization, followed by centrifugation at 20,000 rpm for 5 min at room temperature. Subsequently, 200 µL of the resulting supernatant was applied to Phenomenex^®^ Novum 1 cc Supported Liquid Extraction cartridges (Phenomenex, Vaerloese, Denmark) and allowed to absorb under vacuum. After a 5 min static incubation to facilitate analyte partitioning, the analytes were eluted in two successive steps using 600 µL of a dichloromethane/ethyl acetate mixture (80:20, *v*/*v*) under a gravity flow. The combined eluates were evaporated to dryness under a gentle stream of nitrogen at 35 °C. The dry residues were reconstituted in 300 µL of water/methanol (70:30, *v*/*v*) containing 0.05 mM ammonium fluoride. Following vortex mixing, the reconstituted extracts were transferred to glass autosampler vials fitted for subsequent instrumental analysis. The final net dilution factor introduced by the sample preparation procedure was calculated to be 3.

### 2.6. Analytical Method Validation

Validation followed the European Medicines Agency (EMA) bioanalytical method validation guidelines [17]. The parameters that were assessed included linearity, a lower limit of quantification (LLOQ), intra- and inter-assay precision and accuracy, recovery, matrix effect, analyte carryover, and stability over time. Precision and accuracy were tested at four concentration levels as follows: LLOQ, low, medium, and high. LLOQ acceptance required a <±20% deviation from nominal values, while other levels were accepted within <±15%. Linearity was determined by calibration curves with correlation coefficients (r^2^) ≥ 0.98. Stability testing involved evaluating the sample’s integrity following three freeze–thaw cycles at −20 °C, 24 h exposure at room temperature, and prolonged storage at 4 °C and −20 °C for 5 and 9 days, respectively. A summary of compliance with the EMA bioanalytical method validation guidelines is reported in Table A1.

#### 2.6.1. Calibration Curve Preparation

A primary stock solution (10 µg/mL) of cortisol was dissolved in methanol and stored at −20 °C. Calibration standards spanning from 0.1 ng/mL to 100 ng/mL were freshly prepared in water/methanol (70:30, *v*/*v*) with 1 ng/mL deuterated internal standards, and were analyzed with each batch.

#### 2.6.2. Sensitivity, Linearity, and Carryover

The LLOQ was defined as the lowest concentration with an RSD < 20% and a signal-to-noise ratio of >10, based on repeated measurements. Linearity was confirmed via ten-point calibration curves that were analyzed over three non-consecutive days. Carryover was assessed by injecting three blanks following the highest standard; no residual analyte signal was detected above 20% of the LLOQ.

#### 2.6.3. Accuracy, Precision, Recovery, Matrix Effect, and Selectivity

Method performance was evaluated using pooled urine from six dogs (mixed breeds), spiked with cortisol at LLOQ (0.1 ng/mL), low (0.3 ng/mL), medium (50 ng/mL), and high (100 ng/mL) concentrations. Each level was analyzed in quintuplicate on three separate days to assess its intra- and inter-day variabilities. Precision (expressed as CV%) and accuracy (percentage deviation from nominal) met the EMA criteria. The pooled urine sample was stored at −20 °C during the three separate days.

Recovery was calculated by comparing the analyte responses in extracted urine samples versus non-matrix standards. While urine samples were extracted as previously described, non-matrix standards were prepared by spiking the analyte and internal standard into a mixture of water/methanol (70:30, *v*/*v*) with 0.05 mM ammonium fluoride in order to achieve the desired concentration without any biological interference. The matrix effects were evaluated by spiking post-extraction blank urine and comparing the analyte responses to those in neat solvent. Both the recovery and matrix effects were acceptable when the CV% values were <20%, confirming method robustness and minimal ion suppression or enhancement.

#### 2.6.4. Stability Assessment

Stability studies were performed to evaluate the robustness of the analytes under various storage and handling conditions. Quality control (QC) samples were prepared at the following three concentration levels: low (corresponding to 3× LOQ), intermediate, and high (approaching the upper LOQ). Stock solutions at concentrations of 0.3, 50, and 100 ng/mL were used for these evaluations. Freeze–thaw stability was assessed over three complete cycles involving storage at −20 °C followed by thawing at room temperature. Benchtop stability was determined by leaving the samples at room temperature for 24 h. Additional stability was evaluated after storage at 4 °C and −20 °C for 5 and 9 days, reflecting typical storage conditions and the maximum expected duration before analysis in this study. All stability assessments were conducted in triplicate at each concentration level. The results were considered acceptable if the standard deviation and the deviation from nominal (T0) concentration remained within ±15%, in accordance with the established bioanalytical validation guidelines. Stock and working solution stability was not evaluated because these solutions were freshly prepared before each analytical session, as commonly recommended for steroid analysis.

#### 2.6.5. Reference Interval

The reference intervals for the urinary cortisol-to-creatinine ratio (UCCR) were determined from 48 residual urine samples collected from clinically healthy, client-owned dogs. Dogs were evaluated during routine health checks or preoperative procedures according to the ASVCP reference interval guidelines [18]. To reflect everyday clinical practice, no standardization of sampling time, environment, or collection method was enforced beyond ensuring spontaneous voiding and proper sample handling. In this study, the urinary cortisol-to-creatinine ratio (UCCR) was calculated using cortisol concentrations expressed in nanomoles per liter (nmol/L) and creatinine concentrations in millimoles per liter (mmol/L), resulting in a dimensionless ratio expressed in mol/mol (commonly reported as ×10^−6^).

#### 2.6.6. Statistical Analysis

For the reference interval, statistical analyses were conducted using Reference Value Advisor (V2.1), a freeware statistical tool for Microsoft Excel (2016) that is designed to support clinical laboratory reference interval estimation [19]. Outlier detection was performed using Dixon–Reed, while the data distribution was assessed using Shapiro–Wilk and Anderson–Darling tests (significance threshold set at *p* ≤  0.01). Intervals were calculated using a non-parametric percentile method with bootstrap resampling, as recommended by the IFCC-CLSI C28-A3 guidelines for sample sizes below 120 observations [20]. For a method comparison, Pearson’s correlation coefficient (r), Bland–Altman difference plots with mean, difference, and limits of agreement, and Passing–Bablok regression analysis were performed using MedCalc for Windows, version 23.3.7 (MedCalc Software, Ostend, Belgium).

## 3. Results

### 3.1. Method Validation

A calibration curve was constructed over the range of 0.1 to 100 ng/mL using standard solutions prepared in water/methanol (70:30, *v*/*v*) containing 0.05 mM ammonium fluoride. The calibration range was established based on reported urinary cortisol concentrations in dogs under both physiological and pathological conditions, ensuring the assay’s sensitivity and accuracy for both normal and elevated cortisol concentrations. This provides reliable quantification across a broad spectrum of clinical scenarios. Linear regression analysis was performed without forcing the intercept, applying a 1/x^2^ weighting factor. All calibration points met the acceptance criteria, with back-calculated concentrations deviating by no more than ±15% from the nominal values. The coefficient of determination (R^2^) consistently exceeded 0.99, demonstrating excellent linearity across the tested range. The limit of detection (LOD) was established at 0.05 ng/mL, while the lower limit of quantification (LLOQ) was defined at 0.1 ng/mL, meeting the performance requirements of a signal-to-noise ratio above 10 and a relative standard deviation (RSD) below 15% across five replicates. Method precision and accuracy were evaluated at the following four concentration levels: LLOQ, low (approximately 3× LLOQ), medium (50% of the calibration range), and high (100% of the calibration range). Inter-run accuracy ranged from 87.7% to 105.5%, and the coefficients of variation remained below 15% at all tested levels, including the LLOQ. A summary of inter-assay performance, including the mean concentration, bias, recovery, standard deviation, and CV%, is provided in Table 1. The matrix effect was assessed in triplicate and found to be negligible, with an average close to 100%, indicating minimal ion suppression or enhancement. Carryover was evaluated following the injection of a 100 ng/mL sample; analyte signals in subsequent blank injections remained below 20% of the LLOQ, and the internal standard carryover was less than 5%, confirming the robustness of the method.

#### 3.1.1. Selectivity

**Figure 1:**
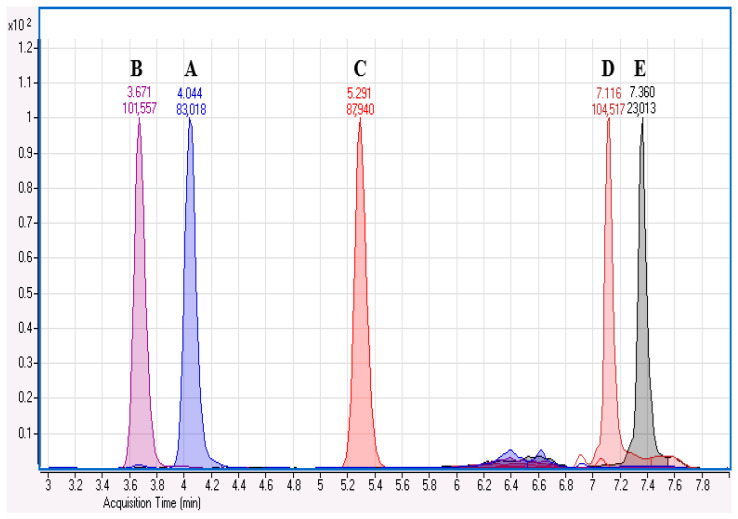
Chromatographic separation of analytes with acquisition from minutes 3 to 8. The figure illustrates the chromatographic resolution of urinary cortisol (A) from both endogenous and exogenous compounds. Cortisol is baseline-separated from the endogenous steroid cortisone (B), as well as from exogenously administered compounds such as methylprednisolone (C), trilostane (D), and dexamethasone (E), confirming the method’s selectivity.

To evaluate potential interferences from exogenous compounds commonly administered in veterinary settings, a selectivity test was performed as part of the method validation. The objective was to assess whether the presence of structurally related synthetic steroids could result in signal interference and, consequently, an overestimation of cortisol levels in real urine samples. For this purpose, cortisone, trilostane, methylprednisolone, and dexamethasone were selected as representative interfering analytes. A blank urine sample was extracted and subsequently spiked with known concentrations of each compound. Retention times were compared to those of the target analyte, and the presence of at least one qualifier ion was required to confirm potential co-elution. The results demonstrated that cortisol was chromatographically resolved from all tested compounds, with distinct retention times and qualifying transitions, thereby confirming the method’s selectivity in the presence of these exogenous steroids (Figure 1).

#### 3.1.2. Stability

Stability testing was conducted using quality control (QC) samples at the following three concentration levels: low (2× LOQ), intermediate, and high (near the ULOQ). Freeze–thaw stability was evaluated over three cycles at −20 °C and at room temperature. Benchtop stability was assessed after 24 h at room temperature, while additional stability evaluations were examined following storage for 5 and 9 days at 4 °C and −20 °C. Concentrations at all QC levels remained within the predefined acceptance limits throughout freeze–thaw cycles, 24 h room temperature exposure, and prolonged storage at both 4 °C and −20 °C (Table 2).

#### 3.1.3. Method Comparison

**Figure 2:**
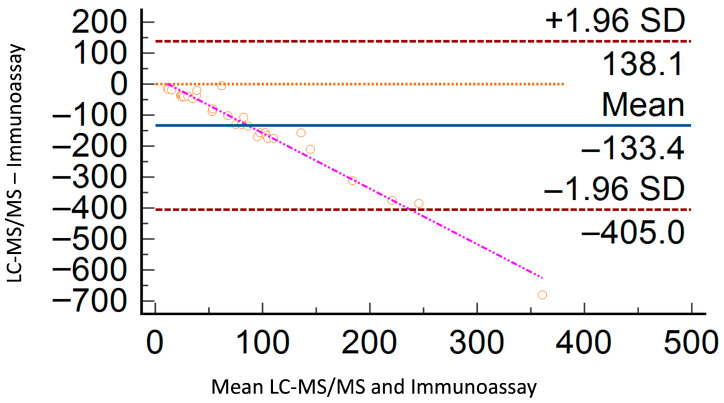
Bland–Altman plot. The mean of the LC-MS/MS measurement and the immunoassay measurement for each sample is plotted on the X axis. The difference between the LC-MS/MS measurement and the immunoassay measurement for each sample is plotted on the Y axis. Orange dotted line = line of identity; blue solid line = mean of differences; red dashed lines: lower and upper limits of agreement; pink dash-dotted line = regression line; orange circles = measured urinary cortisol concentrations.

**Figure 3:**
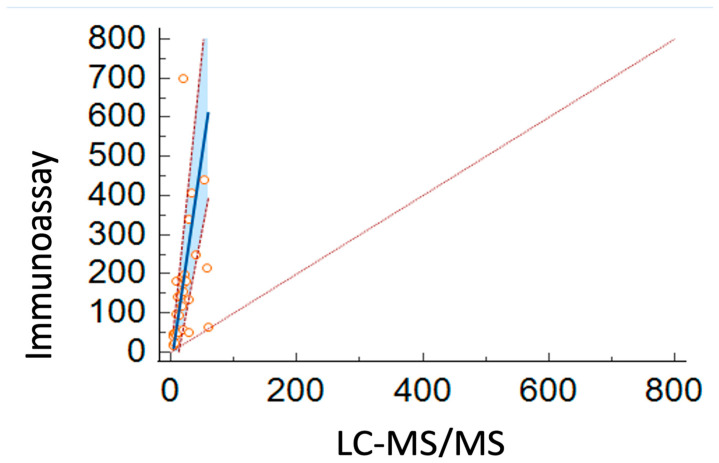
Passing–Bablok regression plot. Red dotted line = line of identity (y = x); solid blue line = regression line; red dashed lines = 95% confidence interval; light blue = confidence interval; orange circles = measured urinary cortisol concentrations.

To evaluate the performance of both methods, the cortisol concentrations obtained from the same samples used to establish the reference ranges were considered. Cortisol values were initially measured in ng/mL and subsequently converted to nmol/L using a correction factor of 2.76. For the immunoassay-based measurements, the median cortisol concentration was 135.6 nmol/L (range: 17.5 nmol/L–700.8 nmol/L). For LC-MS/MS, the median was 16.7 nmol/L (range: 4.3 nmol/L–59.6 nmol/L). A correlation index was calculated for each sample that was analyzed by both methods. A Passing–Bablok and Bland–Altman plots were generated to visualize the relationship between the two methods. The Pearson correlation coefficient (r) between LC-MS/MS and the immunoassay method was 0.71, indicating a moderate positive correlation and supporting the use of Passing–Bablok regression over linear regression. The Bland–Altman difference plot, including the mean difference, is shown in Figure 2, while the Passing–Bablok regression plot is presented in Figure 3. The detailed results of both the Bland–Altman and Passing–Bablok regression analyses are provided in Table 3.

### 3.2. Reference Interval

A total of 48 client-owned dogs were enrolled in the study. Voided urine samples were collected by owners. The mean age of the population was 94.8 months (±43.3 SD), and the mean body weight was 21.2 kg (±13.6 SD). In Table A2, detailed demographic and sampling information for all animals included in the study are reported.

**Figure 4:**
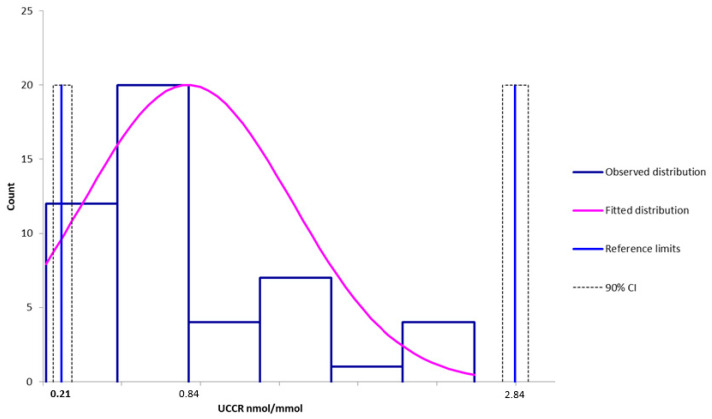
Distribution and reference intervals of UCCR generated using Reference Value Advisor (V2.1) software [19]. CI stands for confidence interval.

The distribution of UCCR was assessed using the Shapiro–Wilk and Anderson–Darling tests, both indicating significant deviations from normality (*p* < 0.01), confirming a non-Gaussian distribution. The UCCR had a median of 0.84 × 10^−6^ (min 0.17 × 10^−6^; max 2.89 × 10^−6^). Given the non-Gaussian distribution of the UCCR values, a non-parametric approach combined with bootstrap resampling was used to derive a reliable reference interval. One possible outlier was detected according to the Dixon method; however, in accordance with the IFCC-CLSI C28-A3 guidelines [20], and unless this value is known to be aberrant, emphasis was placed on retaining rather than excluding it. The sample size was deemed sufficient to compute a non-parametric reference interval, which was estimated to be 0.21–2.84 × 10^−6^. The confidence intervals (CI) for the reference limits were calculated using a bootstrap method. The 90% CI of the lower reference limit was 0.17–0.39 × 10^−6^ while the 90% CI of the upper reference limit was 2.43–2.89 × 10^−6^ (Figure 4).

## 4. Discussion

This study provides an analytical validation according to EMA guidelines for the measurement of urinary free cortisol concentration via LC-MS/MS in dogs and a corresponding reference interval for UCCR. The method showed excellent analytical performance at four different concentrations spanning the reportable range. In particular, accuracy (percent deviation from nominal) was 87–105%, precision (CV%) was 2.39–9.45%, and bias was −12.3–5.53%, meeting the EMA acceptance criteria at each point. Stability over time and freeze–thaw cycles was satisfactory, with tolerable variations under all tested conditions. Moreover, the method demonstrated a good specificity for cortisol and the ability to reliably measure cortisol when cortisone, methylprednisolone, trilostane, and dexamethasone are present. The use of mass spectrometry has been previously used to measure cortisol and its metabolites in the urine of healthy and diseased dogs; however, a thoroughly assessment of the analytical performances of these methods in dogs was not provided [21,22,23].

LC-MS/MS is a versatile analytical technology that has significantly enhanced clinical diagnostics. It has enhanced both the quality and throughput of various diagnostic assays, including those for steroids, amino acids, vitamins, peptides, proteins, neurotransmitters, cancer biomarkers, as well as therapeutic drug monitoring and toxicological assessments [24]. However, the development of LC-MS/MS clinical assays can be complex, requiring careful management of chemical, physical, biological, and infrastructure challenges to ensure accurate test results. With few exceptions, diagnostic LC-MS/MS testing, such as the validation presented in this study, is fully developed and implemented in-house. Laboratory personnel are typically responsible for preparing all testing materials, including calibration standards and mobile phases [25]. Both the assays and the technical expertise are usually developed internally by the diagnostic laboratories themselves [24].

Our study demonstrates the advantages of LC-MS/MS in measuring UCCR, providing a more accurate assessment of cortisol levels. The reference range we report was lower than that obtained with conventional immunoassays, and likely attributable to antibody cross-reactivity with cortisol metabolites. Recent reference intervals for UCCR in healthy dogs vary considerably, depending on the analytical method or assay kit used and physiological factors such as stress or sampling conditions [4,6,26,27]. Differences between methods may reflect the use of an immunoassay originally developed for human serum and not specifically validated for canine urine, which could increase cross-reactivity with cortisol metabolites. This holds true even when considering the known negative bias of current veterinary cortisol assays compared to previous-generation reagents. It is well documented that UFC levels are substantially higher when measured using various immunoassays compared to LC-MS/MS technology, emphasizing the importance of updating and validating reference intervals for UFC using LC–MS/MS [28,29]. This discrepancy is primarily due to cross-reactions with closely related structures [30]. Moreover, the use of the internal standard is a critical component of the LC-MS/MS method employed. This approach effectively mitigates matrix effects and compensates for potential analytical variability, thereby enhancing the accuracy and reproducibility of the results. In complex analytical settings such as this, the systematic inclusion of an internal standard represents an essential advantage for ensuring overall method reliability. In our study, although a strong correlation was observed between the two methods, both proportional and systematic biases were detected. In particular, Passing–Bablok regression indicated a constant bias, as the 95% CI of the y-intercept did not include 0. Similarly, a proportional bias was evident since the 95% CI of the slope did not include 1. Moreover, visual inspection of the Bland–Altman plot revealed a downward trend in the regression line of the differences, suggesting a negative proportional bias of the LC–MS/MS method relative to the immunological method. Such positive bias in immunoassay results could potentially influence clinical interpretations in borderline cases, if confirmed in clinical settings. The LC–MS/MS method demonstrated excellent analytical performance and showed analytical strengths, particularly in terms of selectivity.

In an available study using LC-MS/MS in dogs, although limited to those receiving trilostane treatment, the UCCR values obtained with LC-MS/MS were approximately 1/20 of those provided by conventional immunoassay [14]. The ability to measure free cortisol, rather than urinary corticoids, is considered invaluable in human medicine, as it reduces false positives, improves reproducibility, and prevents the overestimation of cortisol levels [31]. Moreover, the balance between cortisol and its metabolites is dynamic and can be influenced by several physiological and pathological factors, including pregnancy, menstrual cycle phase, and non-adrenal diseases, such as hypothyroidism, obesity, collagen disease, and impaired liver function. For instance, patients with obesity-related metabolic syndrome and non-alcoholic fatty liver disease may exhibit increased urinary cortisol metabolites despite having normal urinary cortisol concentrations [32]. This consideration is particularly important in critical care settings, where cortisol metabolism is significantly altered. In such contexts, the assessment of free cortisol alone becomes essential, as demonstrated in both human and canine medicine [12,33]. LC-MS/MS allows rapid identification of animals that are not responding adequately to critical illness, potentially providing life-saving information.

Although the aforementioned study using LC-MS/MS did not demonstrate a significant clinical benefit in trilostane-treated dogs [14], we believe that avoiding the measurement of metabolites or precursors of cortisol could improve the assessment of therapy effectiveness, which still largely relies on uncertain owner evaluations [1].

The collection of urine by voiding is a non-invasive and straightforward task that can be performed by caregivers. While the method of urine collection for UCCR has not been specifically evaluated [34], home sampling is likely to reduce stress-induced false positives when screening for hyperadrenocorticism [35]. The clinical value of UCCR could be significantly improved by adopting a precise and sensitive method, such as LC-MS/MS [7], especially given the limitations of current endocrine tests, which are prone to both false-positive and false-negative results [1].

Despite the highlighted analytical strengths of this methodology, several limitations must be acknowledged. First, our study included a relatively small and homogeneous sample population, which may restrict the transferability of the proposed reference intervals to broader clinical settings. These values should therefore be considered preliminary and confirmed in future studies, including animals with hypercortisolism and hypoadrenocorticism. The comparison with the immunoassay was exploratory and limited to a single platform and a relatively small number of paired samples; although systematic and proportional biases were observed, further studies are needed to determine the clinical relevance of these differences. Moreover, most of the urine samples were collected by cystocentesis, which may have introduced pre-analytical variability in UCCR values. However, it is worth noting that urine sampling via voiding remains a non-invasive, caregiver-friendly method that facilitates at-home collection and reduces stress-related cortisol fluctuations. While the lack of standardized collection could be seen as a confounding factor, it also reflects real-world clinical practice, enhancing the external validity of our findings. Importantly, all urine samples were stored frozen prior to analysis. Although sample freezing is often viewed as a potential limitation due to possible analyte degradation, our results indicate excellent stability of urinary cortisol under these conditions. This aspect is also representative of a real scenario where diagnostic samples must be delivered to the very few laboratories offering LC-MS/MS determinations. This finding also supports the robustness of LC-MS/MS and confirms its suitability for both retrospective analyses and multicentre studies that rely on stored biological materials.

## 5. Conclusions

To our knowledge, this is the first study of a full method validation for urinary cortisol measurement via LC-MS/MS in dogs. This validated LC–MS/MS method provides a robust and selective measurement of urinary cortisol and establishes a reference interval in dogs. The exploratory comparison with a commercial immunoassay highlighted method-dependent differences, supporting the role of LC–MS/MS as a reference approach for future clinical studies. Our results demonstrate that the UCCR values obtained via LC-MS/MS are substantially lower than those reported with the commercial immunoassay system Immulite^®^ 2000 Xpi, underscoring the need for updated reference ranges tailored to this methodology. The use of non-invasively collected urine samples, combined with the proven stability of cortisol in frozen specimens, supports the practical application of this approach in both routine diagnostics and critical care settings. Further prospective studies are needed to validate the diagnostic performance of an LC-MS/MS-based UCCR in diseased populations and to explore the influence of physiological variables such as age, sex, and diet. Overall, our findings support the integration of LC-MS/MS into veterinary endocrine diagnostics as a reliable and precise alternative to traditional immunoassays.

## Figures and Tables

**Table 1:** Accuracy of the determination of cortisol, in terms of the mean detected concentration. CV acceptance criteria for LOQ: ≤20%; CV acceptance for low, medium, and high levels ≤ 15%; EM% acceptance criteria: ≤20%.

Spike Level		Intra-Assay Day 1	Intra-Assay Day 2	Intra-Assay Day 3	Inter-Assay
LOQ (0.1 ng/mL)	Mean (ng/mL)	0.10	0.10	0.10	0.10
	CV (%)	13.3	8.25	6.97	9.45
	Accuracy (%)	96.0	95.3	102.7	98.0
	Bias (%)	−3.99	−4.69	2.71	−1.99
Low (0.3 ng/mL)	Mean (ng/mL)	0.27	0.26	0.26	0.26
	CV (%)	4.48	4.83	4.79	4.70
	Accuracy (%)	89.2	85.7	88.1	87.7
	Bias (%)	−10.8	−14.3	−11.9	−12.3
Medium (50 ng/mL)	Mean (ng/mL)	50.6	51.5	49.7	50.6
	CV (%)	2.90	3.89	3.44	3.41
	Accuracy (%)	101	102	99.3	101
	Bias (%)	1.29	2.93	−0.66	1.19
High (100 ng/mL)	Mean (ng/mL)	105	106	104	105
	CV (%)	2.72	0.85	3.62	2.39
	Accuracy (%)	105	106	104	105
	Bias (%)	5.84	6.45	4.30	5.53
EM%	Mean	117.8	-	-	-
	CV	2.30	-	-	-

Accuracy (%) = expressed as [(mean observed concentrations/nominal concentration) × 100]; bias (%) = expressed as [(mean observed concentrations − nominal concentration)/(nominal concentration)] × 100; CV (%) = coefficient of variation: (standard deviation/mean) × 100. EM, matrix effect; LOQ, limit of quantification.

**Table 2:** The results of benchtop stability (24 h at room temperature), 5 and 9 days, and freeze–thaw stability (three cycles).

Stability Type	Mean, ng/mL	Standard Deviation, ng/mL	Percentage Deviation from T0
T0	0.21	0.01	-
	53.9	8.27	-
	114	10.2	-
Benchtop (24 h)	0.23	0.02	8.62
	50.9	3.94	−5.54
	108	4.74	−5.38
Refrigerated (5 days)	0.23	0.02	9.44
	55.5	3.61	3.07
	112	3.86	−1.99
Frozen (5 days)	0.22	0.02	6.78
	47.2	1.97	−12.4
	100	4.95	−11.1
Refrigerated (9 days)	0.24	0.01	13.6
	57.7	0.33	7.11
	119	4.56	3.68
Frozen (9 days)	0.24	0.01	12.1
	54.3	0.22	0.84
	106	3.39	−7.45
Freeze–thaw	0.23	0.01	9.33
	58.9	1.23	0.56
	102	6.57	−7.80

**Table 3:** Bland–Altman and Passing–Bablok regression data. 95% CI (95% confidence interval); LoA (limit of agreement).

		Value	95% CI
Bland–Altman	Mean	−133	−183 to −84
	Lower LoA	−405	−490 to −320
	Upper LoA	138	53–222
Passing–Bablok	Intercept	−38.7	−105.4 to −61
	Slope	10.9	8.4–15.5

## Data Availability

The dataset is available on request from the authors.

## Appendix A

**Table A1:** Summary of compliance with EMA bioanalytical method validation guidelines.

Validation Parameter	Assessed	Description of Assessment
Selectivity	Yes	Six individual blank urine samples and a zero standard were evaluated for interferences
Matrix effect	Yes	Post-extraction addition method: comparison between extracted and neat standards
Accuracy	Yes	Evaluated at LLOQ, low, medium, and high QC levels (*n* = 6 replicates per level)
Precision (intra- and inter-day)	Yes	Assessed at LLOQ, low, medium, and high QC levels across three validation days
Recovery	Yes	Determined at low, medium, and high QC concentrations
Calibration curve	Yes	10 non-zero calibration levels (0.1–100 ng/mL), r^2^ > 0.99
Lower Limit of Quantification (LLOQ)	Yes	0.1 ng/mL (S/N > 10; accuracy and precision within EMA acceptance criteria)
Carryover	Yes	Evaluated by the injection of a blank sample after the ULOQ standard
Stability—processed samples	Yes	24 h at room temperature; 9 days at 4 °C; 9 days at −20 °C and 3 freeze–thaw cycles
Stock solution stability	No	Not assessed; stock solutions freshly prepared before each batch as recommended
Working solution stability	No	Not assessed; working solutions freshly prepared before each batch as recommended

**Table A2:** Patient demographics and sampling information.

Age (Months)	Sex	Weight (kg)	Breed	Reason	Sampling Method
108	Intact male	20	Border Collie	Odontoiatric procedure	Cystocentesis
161	Neutered male	4.8	Mixed	Clinical examination	Cystocentesis
141	Spayed female	9.2	Italian Greyhound	Clinical examination	Free Catch
194	Neutered male	2.8	Chihuahua	Clinical examination	Cystocentesis
57	Spayed female	18.3	Siberian Husky	Pre-neutering evaluation	Cystocentesis
81	Intact male	47.3	Romanian Carpathian Shepherd Dog	Clinical examination	Cystocentesis
78	Intact male	14.2	Beagle	Clinical examination	Cystocentesis
59	Spayed female	18.1	Border Collie	Clinical examination	Free Catch
76	Intact male	9.3	Mixed	Clinical examination	Cystocentesis
113	Neutered male	23	Australian Shepherd	Pre-anaesthetic evaluation	Free Catch
56	Intact male	24	Mixed	Clinical examination	Cystocentesis
183	Neutered male	9.1	Mixed	Clinical examination	Cystocentesis
104	Intact male	39	Saarloos Wolfdog	Clinical examination	Cystocentesis
86	Intact male	15.4	Shiba Inu	Clinical examination	Free Catch
113	Intact male	25.5	Australian Cattle Dog	Pre-anaesthetic evaluation	Free Catch
43	Intact male	12.5	Collie	Clinical examination	Cystocentesis
134	Neutered male	16.2	Border Collie	Clinical examination	Free Catch
142	Spayed female	30.3	Labrador Retriever	Clinical examination	Free Catch
96	Spayed female	28.3	Mixed	Clinical examination	Free Catch
54	Spayed female	15	Lagotto Romagnolo	Clinical examination	Cystocentesis
89	Intact male	40	Saarloos Wolfdog	Clinical examination	Cystocentesis
54	Spayed female	53	Basset Hound	Estrus monitoring	Cystocentesis
49	Intact female	5.9	Pekingese	Clinical examination	Cystocentesis
91	Intact male	33.8	Czechoslovakian Wolfdog	Odontoiatric procedure	Free Catch
75	Intact female	9	Cavalier King Charles Spaniel	Odontoiatric procedure	Cystocentesis
45	Spayed female	7.8	West Highland White Terrier	Pre-neutering evaluation	Cystocentesis
80	Spayed female	6.6	Basset Hound	Odontoiatric procedure	Cystocentesis
49	Intact female	3.9	Toy Poodle	Pre-neutering evaluation	Cystocentesis
82	Intact female	65	Great Dane	Odontoiatric procedure	Free Catch
50	Intact female	12.3	Mixed	Pre-neutering evaluation	Cystocentesis
91	Spayed female	7.3	Miniature Schnauzer	Clinical examination	Cystocentesis
50	Intact female	31.6	Labrador Retriever	Clinical examination	Free Catch
67	Intact male	22.7	Mixed	Clinical examination for respiratory signs	Cystocentesis
112	Spayed female	7.4	Dachshund	Clinical examination	Cystocentesis
50	Intact female	14.6	Lagotto Romagnolo	Pre-neutering evaluation	Cystocentesis
93	Spayed female	13.9	Mixed	Clinical examination	Cystocentesis
71	Intact female	24	Chow Chow	Pre-neutering evaluation	Cystocentesis
143	Neutered male	23.7	Mixed	Odontoiatric procedure	Cystocentesis
80	Intact female	18.3	Mixed	Pre-anaesthetic evaluation	Free Catch
87	Spayed female	53.3	Great Dane	Pre-anaesthetic evaluation	Cystocentesis
27.1	Spayed female	51	German Shorthaired Pointer	Pre-neutering evaluation	Cystocentesis
160	Intact female	21.7	Shetland Sheepdog	Odontoiatric procedure	Cystocentesis
201	Neutered male	7	Jack Russell Terrier	Clinical examination	Cystocentesis
61	Intact female	18	Lagorai Shepherd	Odontoiatric procedure	Free Catch
34	Intact female	22	Standard Poodle	Clinical examination	Free Catch
76	Intact female	18.7	Standard Poodle	Clinical examination	Free Catch
123	Intact male	27.3	Standard Poodle	Clinical examination	Free Catch
169	Spayed female	20	Standard Poodle	Clinical examination	Free Catch
